# J-wave syndrome potentially exacerbated by therapeutic hypothermia

**DOI:** 10.1093/omcr/omac021

**Published:** 2022-03-16

**Authors:** Masahiro Kashiura, Yuichi Hamabe, Takashi Moriya

**Affiliations:** Department of Emergency and Critical Care Medicine, Saitama Medical Centre, Jichi Medical University, Saitama, 330-8503, Japan; Tertiary Emergency Medical Centre, Tokyo Metropolitan Bokutoh Hospital, Tokyo, 130-8575, Japan; Department of Emergency and Critical Care Medicine, Saitama Medical Centre, Jichi Medical University, Saitama, 330-8503, Japan

**Figure 1:**
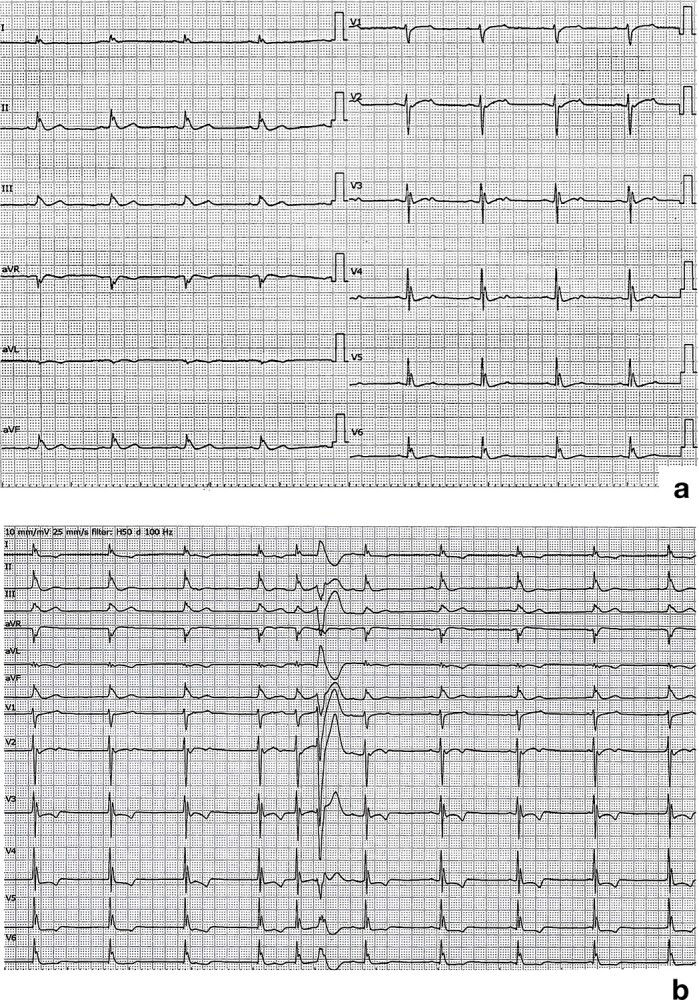
**a)** Electrocardiogram recorded upon admission shows J waves in leads I, II, III, aVR, aVF, and V3–6. b) Electrocardiogram after induced hypothermia shows further elevation of J-waves with multifocal ventricular premature complexes.

A 46-year-old man with no history of syncope presented with sudden cardiac arrest. Prior to arrival, he had received bystander cardiopulmonary resuscitation for 5 min and defibrillation with an automated external defibrillator. Spontaneous circulation had returned while at the scene. However, he was unconscious upon admission to the hospital, where an electrocardiogram (ECG) revealed J-wave elevation over a wide range of leads (I, II, III, aVR, aVF, and V3–6; Fig. 1a). His body temperature (36.8°C) and other vital signs were within the normal limits.

Emergency coronary angiography findings showed no significant coronary artery stenosis. After we induced therapeutic hypothermia (34°C) [1], ECG showed further elevation of J-waves with multifocal ventricular premature complexes (PVCs) (Fig. 1b). Furthermore, the patient experienced recurrent episodes of ventricular fibrillation (VF). Amiodarone, lidocaine, magnesium, and landiolol were ineffective at resolving the VF storm; however, isoproterenol was effective [2]. A Brugada-type electrocardiogram pattern was never observed. Thus, we diagnosed the patient with J-wave (early repolarisation) syndrome. The differential diagnosis of J-wave syndrome includes intraventricular conduction delay-induced end QRS notch syndrome as well as Brugada syndrome [3].

In our patient’s case, therapeutic hypothermia negatively impacted J-wave syndrome [4]. After performing catheter ablation for the PVCs that were triggering VF and implantation of a cardioverter defibrillator, the patient experienced no further incidences of VF [5].
